# Role of neuritin in retinal ganglion cell death in adult mice following optic nerve injury

**DOI:** 10.1038/s41598-018-28425-7

**Published:** 2018-07-04

**Authors:** Yuriko Azuchi, Kazuhiko Namekata, Tadayuki Shimada, Xiaoli Guo, Atsuko Kimura, Chikako Harada, Atsuko Saito, Kanato Yamagata, Takayuki Harada

**Affiliations:** 1 Visual Research Project, Tokyo Metropolitan Institute of Medical Science, Tokyo, Japan; 2 Department of Environmental Science, Graduate School of Science, Toho University, Chiba, Japan; 3 Synaptic Plasticity Project, Tokyo Metropolitan Institute of Medical Science, Tokyo, Japan

## Abstract

Neuritin is a small extracellular protein that plays important roles in the process of neural development, synaptic plasticity, and neural cell survival. Here we investigated the function of neuritin in a mouse model of optic nerve injury (ONI). ONI induced upregulation of *neuritin* mRNA in the retina of WT mice. The retinal structure and the number of retinal ganglion cells (RGCs) were normal in adult neuritin knockout (KO) mice. *In vivo* retinal imaging and histopathological analyses demonstrated that RGC death and inner retinal degeneration following ONI were more severe in neuritin KO mice. Immunoblot analyses revealed that ONI-induced phosphorylation of Akt and ERK were suppressed in neuritin KO mice. Our findings suggest that neuritin has neuroprotective effects following ONI and may be useful for treatment of posttraumatic complication.

## Introduction

Traumatic optic neuropathy is a common clinical problem that occurs in 0.5–5% of patients with closed head injury^1^. A damage to the optic nerve induces secondary swelling within the optic canal, accompanied by subsequent retinal ganglion cell (RGC) loss and optic nerve atrophy^2^. Although effective treatments are not established, previous studies have shown that neurotrophins, such as brain-derived neurotrophic factor (BDNF), protect RGCs in animal models of optic nerve injury (ONI)^3–5^. In addition, suppression of glutamate neurotoxicity, neuroinflammation, oxidative stress and histone deacetylases (HDACs) may be effective for RGC protection^6–11^. Since the ONI model mimics some aspects of glaucoma, it is also a useful animal model for glaucoma^11^.

Neuritin, also known as candidate plasticity gene 15 (CPG15), was first identified as one of the activity-dependent gene products in the brain^12^. Neuritin is an extracellular, glycosylphosphoinositide-linked protein, which can be secreted as a soluble form by various cells including neural and glial cells^13–15^. Neuritin induces neuritogenesis, neurite arborization, neurite outgrowth and synapse formation, which are involved in the development and functions of the central nervous system^15–18^. Loss of neuritin delayed development of the neuropil, including RGC axons and lateral geniculate nucleus, but these deficits were overcome in adult mice^15^. In addition, neuritin is recently thought to be a kind of neurotrophin that regulates neural survival^19^. Exposure of rat cerebellar granule neurons to neuritin markedly induced phosphorylation of Akt, ERK and mammalian target of rapamycin, in part by activating the insulin receptor signaling pathway^19^. Previous studies have reported that Akt activation promotes RGC survival after ONI and activation of the ERK signaling pathway leads to RGC protection in glaucomatous eyes^20,21^. Since the insulin receptor is expressed in the retina including RGCs^22^, in the present study, we examined the effects of ONI on retinal degeneration in neuritin knockout (KO) mice.

## Results

### Upregulation of *neuritin* in the retina following ONI

**Figure 1:**
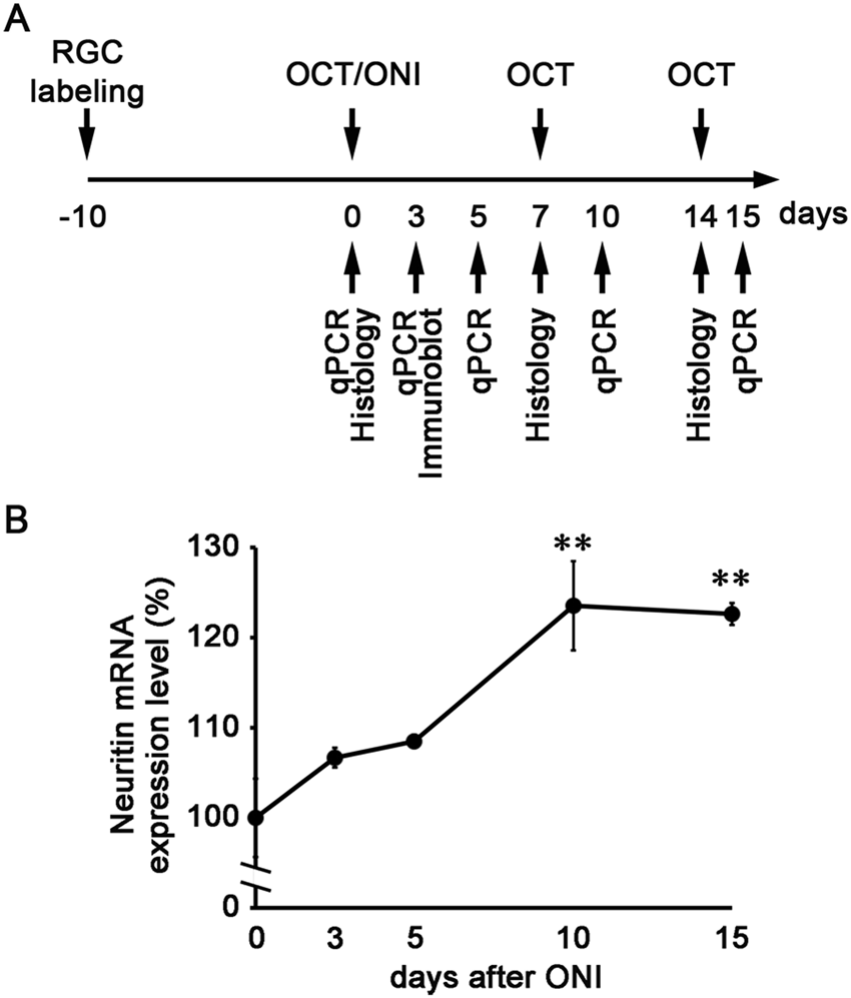
Expression levels of *neuritin* mRNA after ONI in WT mice. **(A)** Experimental timeline. **(B)** mRNA expression levels of *neuritin* in whole retinas at 0, 3, 5, 10 and 15 days after ONI was determined using quantitative real-time PCR analysis. The result is expressed as a percentage of the normal WT mice. Data are presented as means ± S.E.M. *n* = 8 at 0, 3 days after ONI, *n* = 7 at 5 days after ONI, *n* = 6 at 10, 15 days after ONI. **p < 0.01.

We first examined *neuritin* mRNA expression levels in the mouse retina before and after ONI. Quantitative real-time PCR analyses were carried out at 0, 3, 5, 10 and 15 days after ONI (Fig. 1A). *Neuritin* expression was normal at 3 days (106.7 ± 1.1%, *n* = 8; *p* = 0.307) and 5 days (108.5 ± 0.7%, *n* = 7; *p* = 0.157) after ONI, but significantly increased at 10 days (123.6 ± 5.0%, *n* = 6; *p* < 0.0001) and 15 days (122.6 ± 1.2%, *n* = 6; *p* < 0.0001) after ONI compared with normal mice (Fig. 1B). These results suggest that neuritin plays an important role in the retina following ONI.

### Accelerated retinal degeneration after ONI in neuritin KO mice

**Figure 2:**
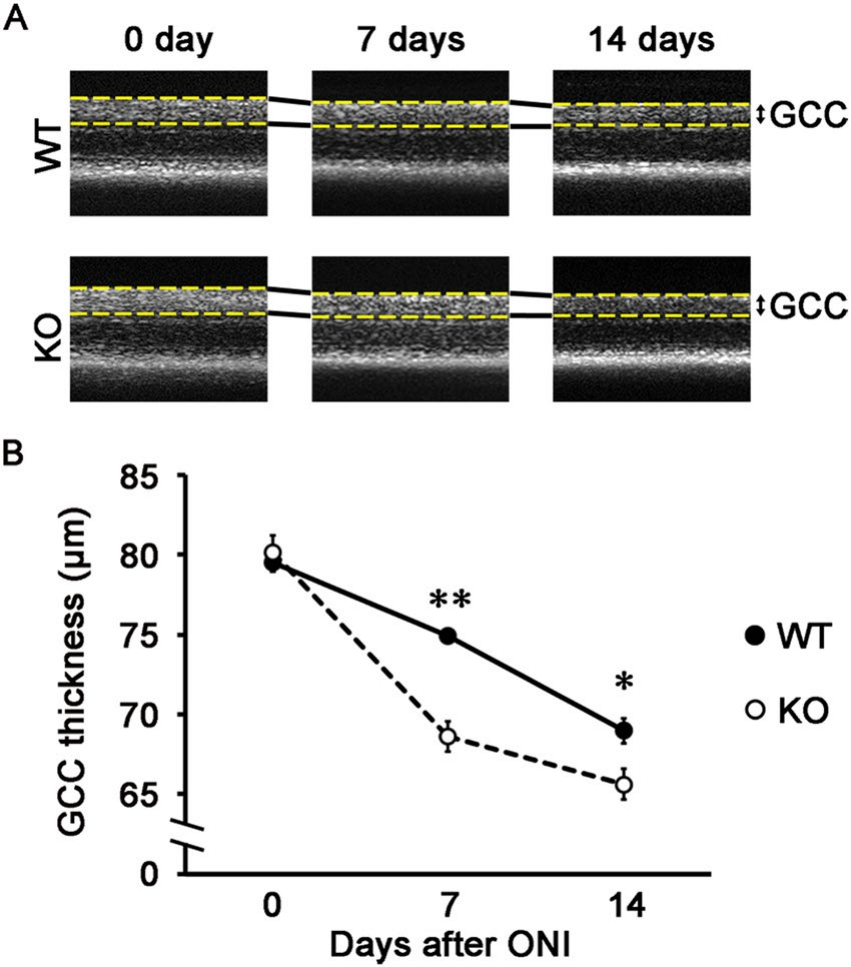
*In vivo* imaging of the retina in WT and neuritin KO mice. **(A)** Representative OCT cross-sectional images of retinas at 0, 7, 14 days after ONI in WT and neuritin KO mice. The dotted yellow lines indicate the ganglion cell complex (GCC). **(B)** Corresponding longitudinal evaluation of the GCC thickness. Data are presented as means ± S.E.M. *n* = 6 per group. *p < 0.05, **p < 0.01.

To examine the functions of neuritin, we next investigated the severity of ONI-induced retinal degeneration in WT and neuritin KO mice. We visualized retinal layers using spectral-domain optical coherence tomography (SD-OCT), a noninvasive imaging technique that is useful for monitoring the changes in retinal structures after injury in living animals^9,23–25^. The SD-OCT images revealed that the mean thickness of the ganglion cell complex (GCC) in neuritin KO mice was significantly decreased compared with WT mice at 7 days after ONI (68.6 ± 0.9 µm vs 74.9 ± 0.3 µm, *n* = 6; *p* < 0.001). A similar difference between neuritin KO and WT mice was found at 14 days after ONI (65.6 ± 0.9 µm vs 69.0 ± 0.8 µm, *n* = 6; *p* = 0.022) (Fig. 2).

**Figure 3:**
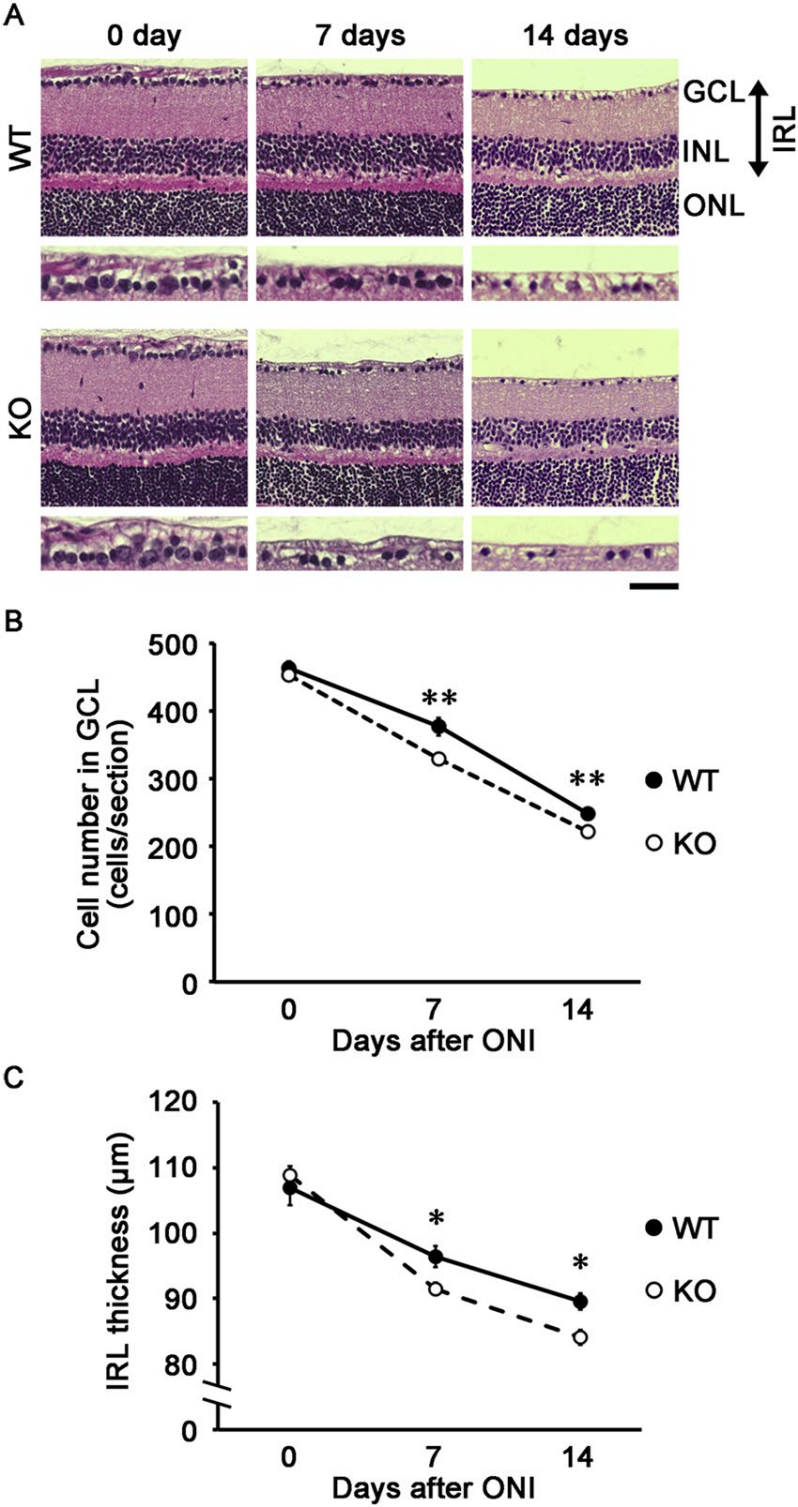
Accelerated retinal degeneration after ONI in neuritin KO mice. **(A)** Representative retinal sections stained with hematoxylin and eosin at 0, 7, 14 days after ONI in WT and neuritin KO mice. Scale bar: 50 and 25 µm in the upper and immediately lower panels, respectively. GCL, ganglion cell layer; INL, inner nuclear layer; ONL, outer nuclear layer; IRL, inner retinal layer. **(B**,**C)** Quantitative analyses of the cell number in the GCL per section (B) and IRL thickness (C). Data are presented as means ± S.E.M. *n* = 6 per group. *p < 0.05, **p < 0.01.

We then examined histopathology of the retina at 7 and 14 days after ONI. The cell number in the ganglion cell layer (GCL) was decreased in both WT and neuritin KO mice following ONI, but the cell loss was more severe in neuritin KO mice compared with WT mice at 7 days (329 ± 6 cells/section vs 377 ± 13 cells/section, *n* = 6; *p* = 0.008) and 14 days (221 ± 5 cells/section vs 248 ± 3 cells/section, *n* = 6; *p* = 0.002) after ONI (Fig. 3A,B). In addition, the thickness of the inner retinal layer (IRL; between the internal limiting membrane and the interface of the outer plexiform layer and the outer nuclear layer) in neuritin KO mice was also decreased compared with WT mice at 7 days after ONI (91.5 ± 1.0 µm vs 96.4 ± 1.6 µm, *n* = 6; *p* = 0.025). A similar difference between neuritin KO and WT mice was also found at 14 days after ONI (84.1 ± 1.2 µm vs 89.6 ± 1.3 µm, *n* = 6; *p* = 0.010) (Fig. 3A,C).

**Figure 4:**
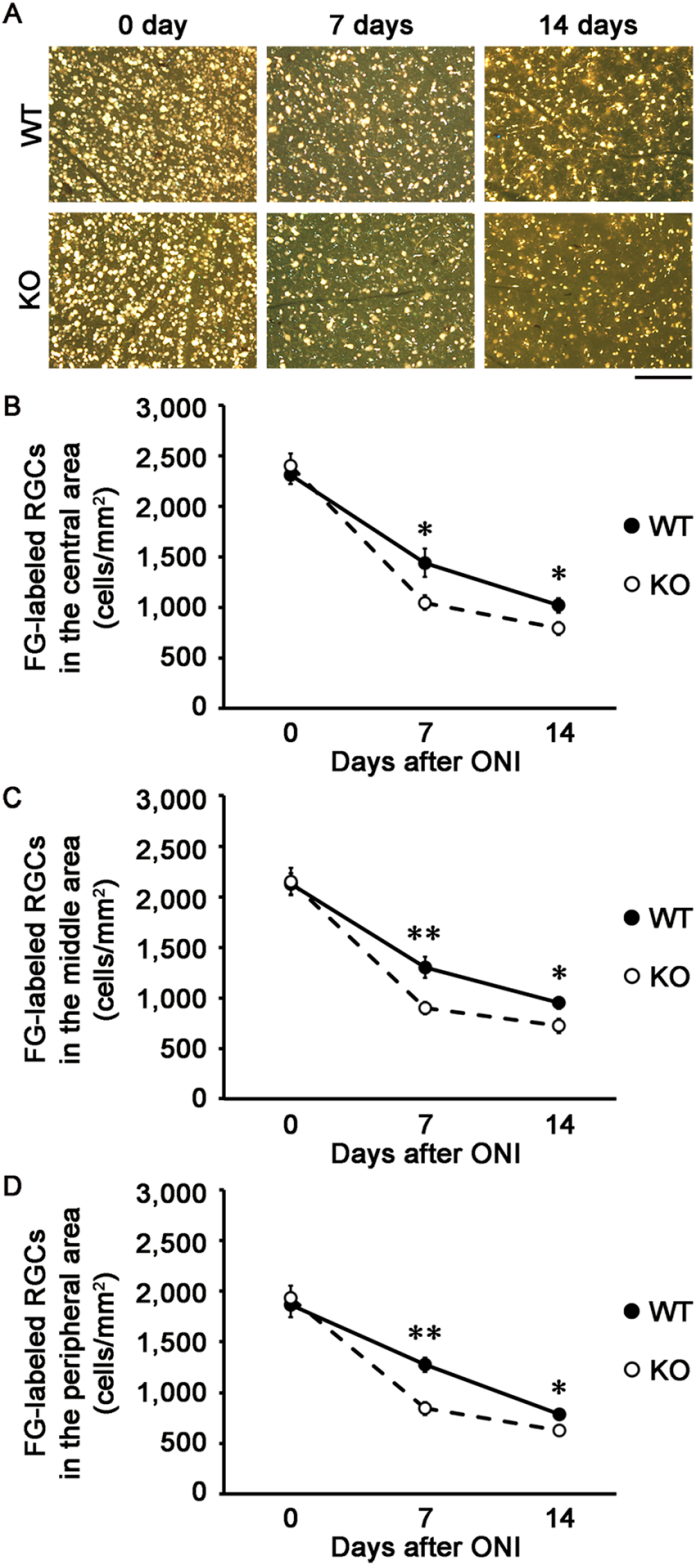
Accelerated RGC loss after ONI in neuritin KO mice. **(A)** Representative images of retrograde-labeled RGCs from the middle areas of the retinas in WT and neuritin KO mice at 0, 7, 14 days after ONI. Scale bar: 100 μm. **(B–D)** Quantification of FG-labeled RGCs in the central (B), middle (C) and peripheral (D) area. Data are presented as means ± S.E.M. *n* = 6 per group. *p < 0.05, **p < 0.01.

Because GCL contains cell types other than RGCs including displaced amacrine cells^26^, we next performed retrograde labeling of RGCs with Fluoro-Gold (FG) and determined the effect of neuritin on RGC survival. Consistent with the results of cell counting in the GCL (Fig. 3B), the RGC number was decreased in neuritin KO mice compared with WT mice at both 7 days (1046 ± 74 cells/mm^2^ vs 1441 ± 142 cells/mm^2^, *n* = 6; *p* = 0.033) and 14 days (790 ± 64 cells/mm^2^ vs 1018 ± 71 cells/mm^2^, *n* = 6; *p* = 0.037) after ONI in the central retina (Fig. 4A,B). In addition, the RGC number was significantly decreased in neuritin KO mice compared with WT mice at 7 days (900 ± 62 cells/mm^2^ vs 1301 ± 104 cells/mm^2^, *n* = 6; *p* = 0.008) and 14 days (724 ± 72 cells/mm^2^ vs 949 ± 58 cells/mm^2^, *n* = 6; *p* = 0.034) after ONI in the middle retina (Fig. 4C). In the peripheral retina, a similar difference between neuritin KO and WT mice was observed at 7 days (843 ± 64 cells/mm^2^ vs 1276 ± 72 cells/mm^2^, *n* = 6; *p* = 0.001) and 14 days (624 ± 47 cells/mm^2^ vs 786 ± 29 cells/mm^2^, *n* = 6; *p* = 0.015) after ONI (Fig. 4D). Taken together, these results suggest that neuritin slows the process of RGC loss all across the retina and retinal degeneration following ONI.

### Effects of neuritin on cell survival signaling in the retina after ONI

**Figure 5:**
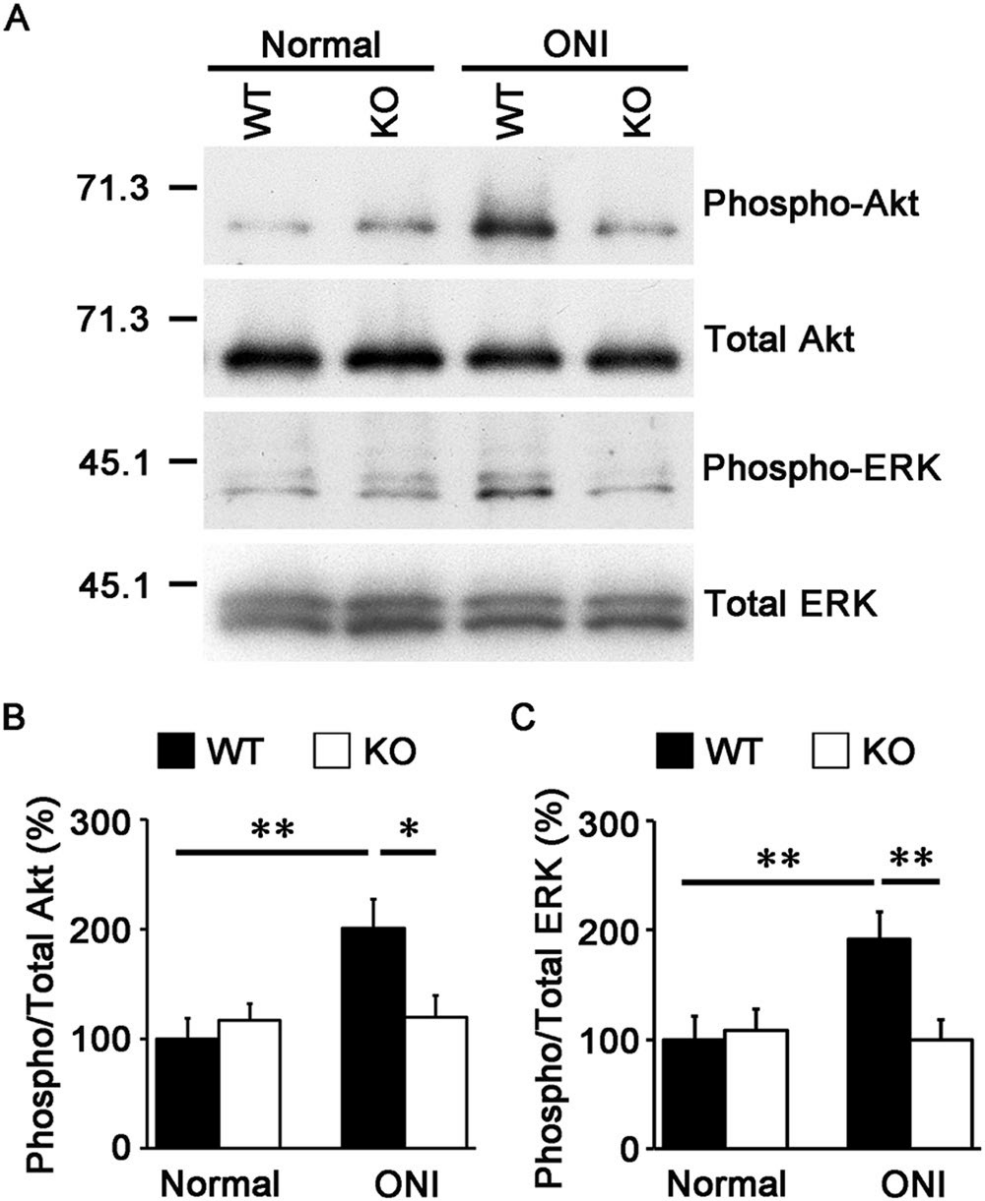
Effects of neuritin on ONI-induced activation of Akt and ERK in the retina. **(A)** Immunoblot analysis of phosphorylated (Phospho-) and total Akt and ERK before and 3 days after ONI in the retina of WT and neuritin KO mice. Full length blot images are presented in Supplementary Figure 3. **(B**,**C)** Relative expression levels of phosphorylated proteins are quantified. The results are expressed as percentage of the normal WT mice and are presented as means ± S.E.M. *n* = 8 per group. *p < 0.05, **p < 0.01.

We also investigated if neuritin has any effects on stimulation of cell survival signaling pathways in the retina following ONI. Previous studies have reported that Akt activation promotes RGC survival after ONI and activation of the ERK signaling pathway leads to RGC protection in glaucomatous eyes^20,21^. We therefore examined the effects of neuritin on ONI-induced activation of the Akt and ERK signaling. Immunoblot analysis revealed that ONI induces expression of phosphorylated (activated) Akt in WT mice (200.1 ± 26.4%, *n* = 8; *p* = 0.004), but not in neuritin KO mice (119.4 ± 20.1%, *n* = 8; *p* = 0.453) (Fig. 5A,B). ONI also activated the ERK signaling in WT mice (191.3 ± 25.6%, *n* = 8; *p* = 0.008), but not in neuritin KO mice (99.6 ± 17.9%, *n* = 8; *p* = 0.370) (Fig. 5A,C). These results suggest that neuritin is associated with activation of Akt- and ERK-mediated cell survival signaling.

## Discussion

In this study, we reported that loss of neuritin accelerates RGC loss and retinal degeneration in adult mice following ONI. We also demonstrated that ONI-induced activation of Akt and ERK, which mediate pro-survival signaling in RGCs^20,21^, were inhibited in neuritin KO mice. Previous studies have reported that neuritin exerts neuroprotective effects by activating the insulin receptor signaling pathway^19^. The insulin receptor is expressed in various cell types in the retina^22,27,28^, and insulin and its receptor system may play a critical role in maintaining neuronal function and survival in the retina by activating Akt and ERK^19,22,29,30^. In addition, insulin-like growth factor-1 (IGF-1), an important factor in retinal development, prevents ONI-induced RGC death^31,32^. Thus, neuritin-insulin receptor-Akt and -ERK pathways in RGCs may be valid therapeutic targets for treatment of posttraumatic complication and glaucoma.

Previous studies have shown that neurotrophins, such as BDNF and neurotrophin-3 (NT-3), and their receptors are involved in the development of visual systems and protection of retinal neurons from various types of injury^3–5,23,24,33–38^. Interestingly, neuritin expression is induced by neuronal activity and by the activity-regulated neurotrophins BDNF and NT-3^13^. A recent study reported that the soluble form of neuritin was secreted from astrocytes in response to an ischemic insult and this could ameliorate the recovery of the ischemia-injured hippocampal neurons^14^. In addition, transcorneal electrical stimulation rescues the axotomized RGCs by increasing IGF-1 production in Müller glial cells^39,40^. Since neuritin and IGF-1 are soluble secreted proteins that can bind to the insulin receptor^19^, in response to neural activity and neurotrophins, neuritin and other trophic factors may be secreted from various cell types and stimulate RGC survival via autocrine and paracrine manners. A recent study supported neuroprotective effects of neuritin by reporting that adeno-associated virus (AAV)-mediated overexpression of neuritin delayed RGC apoptosis, regenerated injured axons, and maintained RGC function following ONI^41^. Similar protective effects of neuritin was reported in a rat model of sciatic nerve injury^42^.

Our present study demonstrated that ONI induces upregulation of *neuritin* mRNA in the retina of C57BL/6 J mice at 10 and 15 days after ONI. On the other hand, in BALB/cJ mice, *neuritin* mRNA displayed a biphasic level of expression with significantly decreased expression from basal levels at 3 and 21 days after ONI and modestly decreased expression at 14 days after ONI^43^. In a rat model of spinal cord injury, *neuritin* mRNA showed significantly reduced expression at 1 day, with subsequent expression recovery between 7 and 14 days after spinal cord injury^44^. The discrepancy may be due to differences in experimental animals, injuries and time points.

We recently reported that some existing drugs are useful for RGC protection. For example, valproic acid (VPA), one of the HDAC inhibitors, protects RGCs from glutamate neurotoxicity and in a mouse model of normal tension glaucoma^24,45^. VPA is also effective for RGC protection after ONI^10^. Interestingly, VPA stimulates productions of nerve growth factor and BDNF in cultured Müller glial cells^24^. These results suggest that VPA may induce neuritin expression by stimulating productions of neurotrophins. Although further *in vivo* studies are required, our findings raise intriguing possibilities for the management of ONI and RGC degeneration by existing drugs such as oral VPA in combination with local application of exogenous neurotrophins and neuritin.

## Methods

### Mice

Experiments were performed using C57BL/6 J mice (CLEA Japan, Tokyo, Japan) or neuritin KO mice (*Nrn1*^tm1.2Ndiv^: stock # 018402, Jackson Laboratory, Bar Harbor, ME, USA)^18^, in accordance with the Tokyo Metropolitan Institute of Medical Science Guidelines for the Care and Use of Animals. Light intensity inside the cages ranged from 100 to 200 lux and a 12 hours light/12 hours dark cycle was maintained. All experiments were approved by the Tokyo Metropolitan Institute of Medical Science. Neuritin gene KO was confirmed by PCR genotyping of mouse tail DNA according to the protocol provided by Jackson Laboratories, which was based on a previous report^16^. A WT forward primer (5′-GTCGCAGCCCAATCTGCATTCC-3′), a neuritin KO forward primer (5′-GCCGTTGTGGTCTTCCAAAGACC-3′), and a common reverse primer (5′-CGGGTTTCCAAAATAATGAGCGAC-3′) were used (Supplementary Figure 1). To further confirm the deletion, another PCR of tail genomic DNA was performed. For the *neuritin* exon 2 amplification, a forward primer (5′-GGTCAGTAGTGGGGCAGAGTGGCGGTGATG-3′) and a reverse primer (5′-AAGGGAAACCCAGGGTCAGAGAGGACACTT-3′) were used. For glyceraldehyde-3-phosphate dehydrogenase (*gapdh)* control amplification, a forward primer (5′-TGCACCACCAACTGCTTAG-3′) and a reverse primer (5′-GGATGCAGGGATGATGTTC-3′) were used (Supplementary Figure 2).

### Retrograde RGC labeling and optic nerve injury

Mice were deeply anesthetized with isoflurane (Intervet, Tokyo, Japan), placed on stereotaxic frame, and received an injection of 2 µL FG (1% in phosphate-buffered saline; Fluorochrome LLC, Denver, CO, USA) into the superior colliculus^23,24^. At 10 days after FG application, mice were anesthetized by intraperitoneal injection of sodium pentobarbital (87.5 mg/kg) before subjected to an ONI procedure. Optic nerves were exposed intraorbitally and crushed at about 0.5 to 1.0 mm from the posterior pole of the eyeball with fine surgical forceps for 5 s^23,25,46^. On 7 and 14 days after ONI, mice were killed by cervical dislocation, eyes were enucleated, and retinas were isolated for whole mount preparation. Retinas were fixed in Zamboni’s fixative (2% paraformaldehyde and 15% picric acid in 0.1 M phosphate buffer) for 20 min, mounted on a slide glass with a mounting medium (Vectashield; Vector Laboratories Inc., Burlingame, CA, USA), and the RGC density was examined with a fluorescent microscope. Six standard areas (0.09 mm^2^) were selected from each retina as follows: one was from the central area (0.1 mm from the optic disc), two were from the middle area (0.8 mm from the optic disc), three were from the peripheral area (1.5 mm from the optic disc)^47^. FG-labeled cells were counted, and the mean number of RGCs per square millimeter was calculated.

### Quantitative real-time PCR

Quantitative real-time PCR was performed using an MyiQ Single-Color Real-Time PCR Detection System (Bio-Rad, Hercules, CA, USA) with a THUNDERBIRD SYBR qPCR Mix (TOYOBO, Osaka, Japan) as described previously^24,33^. Total RNA for PCR was prepared from whole retinas from six to eight different eyes at 0, 3, 5, 10 and 15 days after ONI. Complementary DNA reverse transcribed from total RNA was amplified by using primers specific for *neuritin* (sense: 5′-TCT TAC GGA TTG CCA GGA AG-3′, antisense: 5′-GCT AAA GCT GCC GAG AGA GA-3′) and glyceraldehyde 3-phosphate dehydrogenase (GAPDH; sense: 5′-TGC ACC ACC AAC TGC TTA G-3′, antisense: 5′-GGA TGC AGG GAT GAT GTT C-3′). Data were normalized to the level of GAPDH mRNA.

### Imaging acquisition of SD-OCT

Mice were anesthetized by intraperitoneal injection of sodium pentobarbital and SD-OCT (RS-3000; Nidek, Aichi, Japan) examinations were performed at 0, 7, 14 days after ONI^9,23,25^. To get fundus imaging, polymethyl methacrylate contact lenses optimal for mice (UNICON, Osaka, Japan) were placed on the corneas for prevention of anesthesia-induced cataract progression. A 60-D adaptor lens was placed on the objective lens of the Multiline OCT to focus on the mouse retina. All the images were location matched, scanning vertically through the center of the optic nerve head at 3-disk diameter lengths above the optic nerve head. The mean thickness of the GCC, between the internal limiting membrane and the interface of the inner plexiform layer and the inner nuclear layer, was measured. In this study, the maximum number of B-scans set by the manufacturer (50 for line scans) was used for averaging.

### Histological and morphometric studies

At 0, 7, 14 days after ONI, mice were anesthetized with an intraperitoneal injection of sodium pentobarbital and perfused transcardially with saline, followed by Zamboni’s fixative. Eyes were removed and postfixed in 3% glutaraldehyde solution (3% glutaraldehyde, 9% formaldehyde, 37.5% ethanol, and 12.5% acetic acid in distilled water) for 2 h. Paraffin embedded retinal sections of 7 µm thickness were cut through the optic nerve and stained with hematoxylin and eosin. The extent of retinal degeneration was quantified in two ways^48,49^. First, the number of neurons in the GCL was counted from one ora serrata through the optic nerve to the other ora serrata. Second, in the same sections, the thickness of the IRL was measured.

### Immunoblot analysis

Immunoblotting was performed for whole retina protein extracts from eight different eyes at 0 and 3 days after ONI, as described previously^23,50^. Membranes were incubated with an antibody against Akt (1:1000; Cell Signaling Technology, Beverly, MA, USA), phospho-Akt (1:1000; Cell Signaling), ERK (1:1000; Cell Signaling) or phospho-ERK (1:1000; BD Biosciences, Franklin Lakes, NJ, USA). Primary antibodies binding was detected using horseradish peroxidase-linked anti-mouse IgG or anti-rabbit IgG secondary antibodies (1:1000; Cell Signaling) and visualized by exposing to X-ray film (Advansta, Menlo Park, CA, USA) for 1 min. with Chemi-Lumi One Ultra (Nacalai Tesque, Kyoto, Japan). The band intensities were quantified using the NIH Image program (ImageJ 1.50c4; NIH, Bethesda, MD, USA).

### Statistics

For statistical analysis, we used a two-tailed Student’s *t*-test for comparison of two groups or one-way ANOVA followed by Dunnett’s post hoc test for multiple comparisons, as appropriate. Data are presented as means ± S.E.M. *P* < 0.05 was regarded as statistically significant. JMP version 13.1.0 (SAS Institute Inc., Cary, NC, USA) was used for the statistical analyses.

## Electronic supplementary material

Supplementary Figure
