# Supplementary material for: Role of neuritin in retinal ganglion cell death in adult mice following optic nerve injury
Source: Sci Rep. 2018 Jul 4;8:10132. doi: 10.1038/s41598-018-28425-7 (PMC6031618; doi:10.1038/s41598-018-28425-7)
Supplement: Supplementary file 1 — Supplementary Figure [file 41598_2018_28425_MOESM1_ESM.docx]

Supplementary Information for

**Role of neuritin in retinal ganglion cell death in adult mice following optic nerve injury**

**Yuriko Azuchi,^1,2^ Kazuhiko Namekata,^1,2^ Tadayuki Shimada,^3^ Xiaoli Guo,^1^ Atsuko Kimura,^1^ Chikako Harada,^1^ Atsuko Saito,^2^ Kanato Yamagata,^3^ and Takayuki Harada^1^**

^1^Visual Research Project, Tokyo Metropolitan Institute of Medical Science, Tokyo, Japan.

^2^Department of Environmental Science, Graduate School of Science, Toho University, Chiba, Japan

^3^Synaptic Plasticity Project, Tokyo Metropolitan Institute of Medical Science, Tokyo, Japan.

*Corresponding author: **Kazuhiko Namekata, Ph.D.**

Visual Research Project, Tokyo Metropolitan Institute of Medical Science

2-1-6 Kamikitazawa, Setagaya-ku, Tokyo 156-8506, Japan.

E-mail: namekata-kz@igakuken.or.jp

**
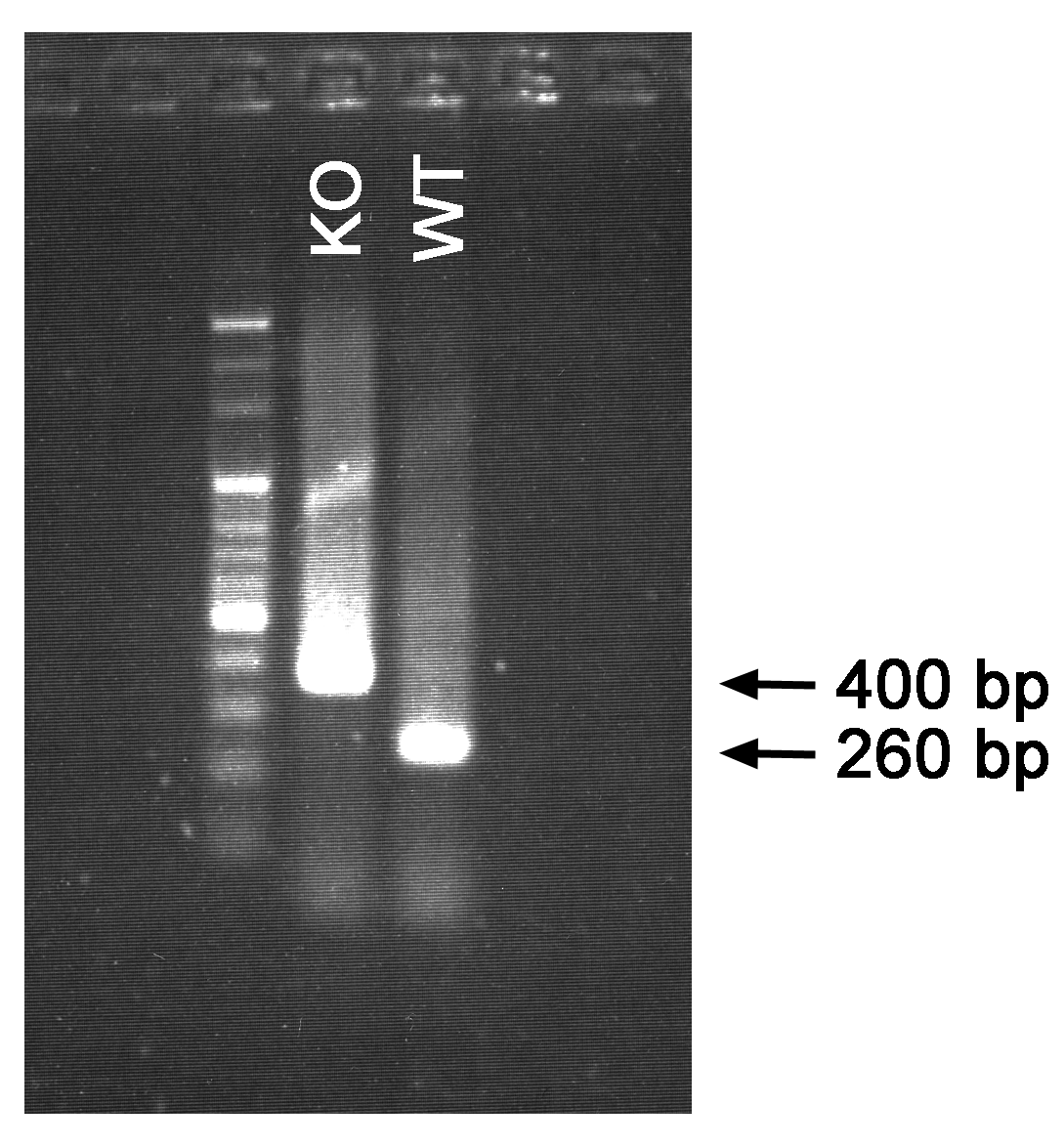
Supplementary figure 1. PCR genotyping of *neuritin* KO mice.**

The amplified 260 bp fragment indicates WT and the amplified 400 bp fragment indicates *neuritin* KO.


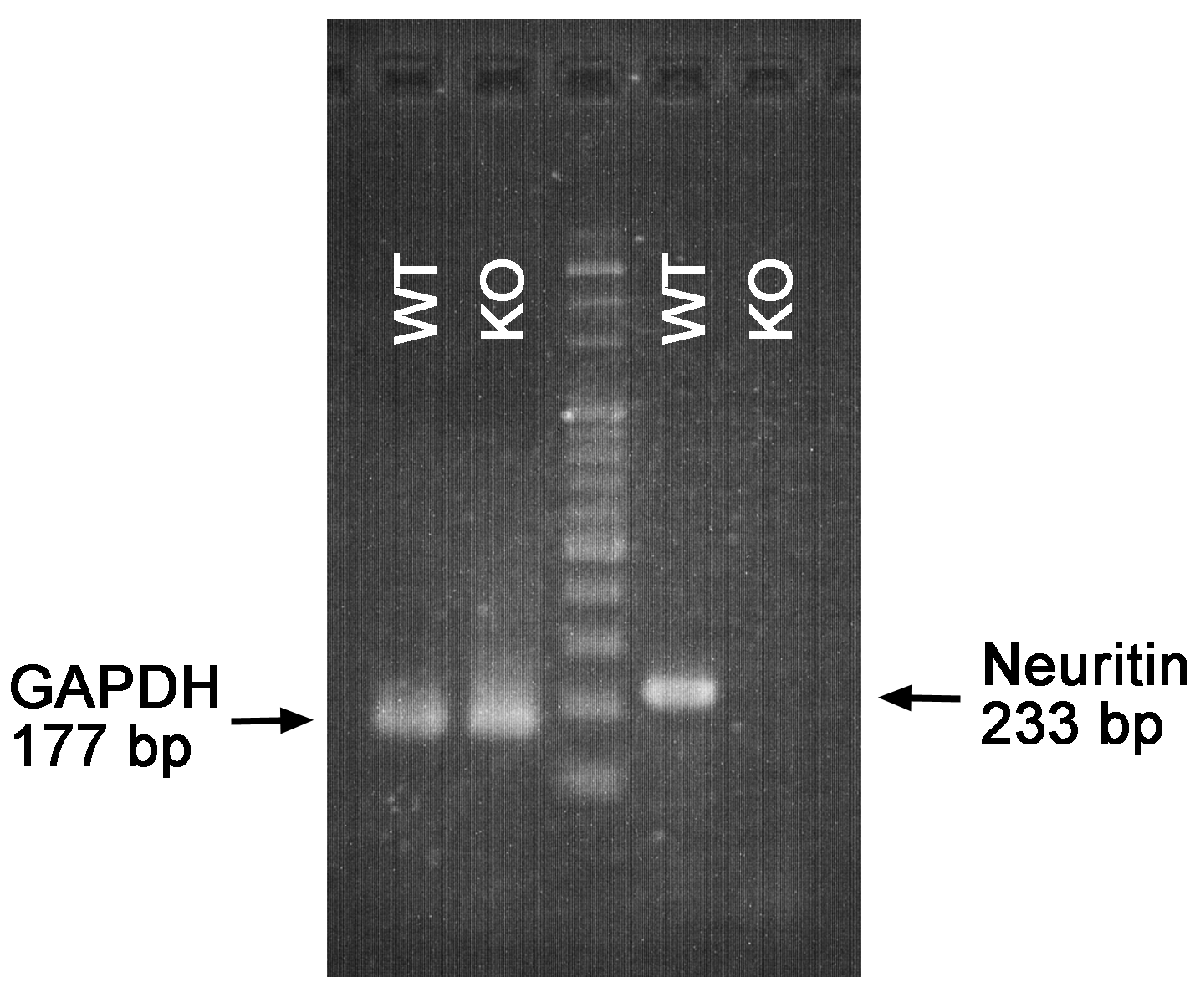
**Supplementary figure 2. The absence of *neuritin* genomic sequence confirmed by PCR of tail genomic DNA.**

The 177 bp fragments were amplified from the GAPDH gene and the 233 bp fragment was derived from the exon2 of the *neuritin* gene.

**
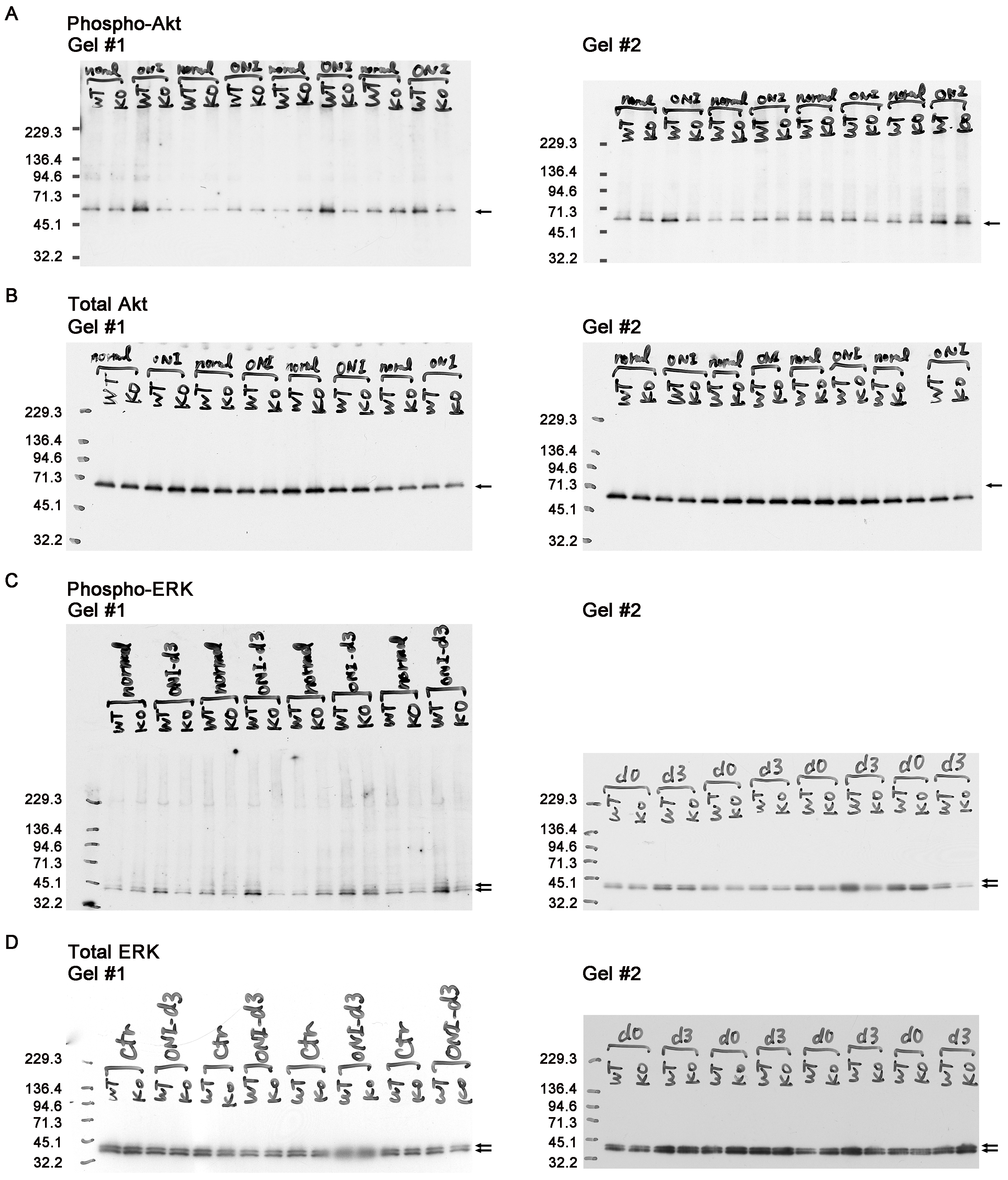
**

**Supplementary figure 3. Original blots for Fig. 5A.**
